# Incidence of acute kidney injury (AKI) and outcomes in COVID-19 patients with and without antiviral medications: A retrospective study

**DOI:** 10.1371/journal.pone.0292746

**Published:** 2023-10-11

**Authors:** Seyed Majid Mousavi Movahed, Hamed Akhavizadegan, Fatemeh Dolatkhani, Samaneh Akbarpour, Seyed Aria Nejadghaderi, Morvarid Najafi, Parmida Sadat Pezeshki, Akram Khalili Noushabadi, Hoomaan Ghasemi

**Affiliations:** 1 Baharloo Hospital, School of Medicine, Tehran University of Medical Sciences, Tehran, Iran; 2 Urology Department, Baharloo Hospital, Tehran University of Medical Sciences, Tehran, Iran; 3 Nephrology Department, Baharloo Hospital, Tehran University of Medical Sciences, Tehran, Iran; 4 Occupational Sleep Research Center, Baharloo Hospital, Tehran University of Medical Sciences, Tehran, Iran; 5 School of Medicine, Shahid Beheshti University of Medical Sciences, Tehran, Iran; 6 Systematic Review and Meta-analysis Expert Group (SRMEG), Universal Scientific Education and Research Network (USERN), Tehran, Iran; 7 School of Medicine, Tehran University of Medical Sciences, Tehran, Iran; 8 Baharloo Hospital, Tehran University of Medical Sciences, Tehran, Iran; Guilan University of Medical Sciences, ISLAMIC REPUBLIC OF IRAN

## Abstract

**Background:**

Acute kidney injury is a complication of COVID-19 and is associated with severity. Despite no specific antiviral treatment strategy, lopinavir/ritonavir and remdesivir have been used. Data on the association between AKI and receiving antiviral agents with outcomes in hospitalized patients with COVID-19 is scarce. We aimed to determine the incidence of AKI and its outcomes in COVID-19 patients with and without antiviral medications.

**Methods:**

We conducted a retrospective study on hospitalized adult patients with SARS-CoV-2 infection in a tertiary center. The primary endpoint was determining mortality, intensive care unit (ICU) admission, and length of hospitalization affected by AKI development using antiviral agents. The logistic regression method was used to explore the predictive effects of AKI and antiviral therapy on composite outcomes (i.e., mortality, ICU admission, and prolonged hospitalization) in four defined groups by AKI development/not and utilizing antivirals/not. We used IBM SPSS version 24.0 software for statistical analysis.

**Results:**

Out of 833 COVID-19 patients who were included, 75 patients were treated with antiviral agents and developed AKI. There was a significant difference in the occurrence of AKI and using antiviral medications (p = 0.001). Also, the group using antiviral agents and the development of AKI had the highest rate of preexisting hypertension (p = 0.002). Of note, the group of patients who used antiviral agents and also developed AKI had the most remarkable association with our composite outcome (p<0.0001), especially ICU admission (OR = 15.22; 95% CI: 8.06–27.32).

**Conclusions:**

The presence of AKI among COVID-19 patients treated with antiviral agents is linked to increased severity and mortality. Therefore, it is imperative to explore preventive measures for AKI development in patients receiving antiviral therapy. Larger-scale randomized controlled trials may be warranted to provide a more comprehensive understanding of these associations.

## Introduction

On March 11, 2020, coronavirus disease 2019 (COVID-19), caused by severe acute respiratory syndrome coronavirus 2 (SARS-CoV-2), was declared a pandemic by World Health Organization (WHO) [1]. Over 770 million confirmed cases of COVID-19, including almost 7 million deaths, have been reported worldwide as of early September 2023 [2]. Although COVID-19 mainly affects the respiratory system and can lead to acute respiratory distress syndrome (ARDS) [3], it is recognized that it can also impact multiple organ systems [4], especially the renal system [5]. Male sex and advanced aging were considered risk factors for severe COVID-19 [6]. Some underlying diseases like thyroid disease and preeclampsia can also increase the risk and severity of COVID-19 [7, 8]. Moreover, patients with comorbidities such as hypertension, diabetes, and cardiovascular disease were more likely to develop severe COVID-19 and death [6, 9].

The SARS-CoV-2, by direct kidney infection, immune responses dysregulation, and second hit phenomenon, can cause acute kidney injury (AKI)- one of the complications of COVID-19 [3, 10]. According to a systematic review and meta-analysis, the incidence rate of AKI in patients with COVID-19 was 8.9% (95% confidence interval (CI): 4.6%, 14.5%) [11]. While it was associated with severity and deaths in which 77% of patients who developed AKI had a severe infection and 52% died [12].

Some of the antiviral agents that can be used for COVID-19 include lopinavir/ritonavir, and remdesivir [13]. Trials from China and the United Kingdom (UK) found no benefit with lopinavir/ritonavir treatment beyond the standard of care [14–16]. Also, another clinical trial showed remdesivir was more effective than placebo in shortening recovery time in hospitalized patients with COVID-19 [17].

In our previous paper, we evaluated the effects of antibiotics on the outcomes of patients with COVID-19 who developed/not developed AKI, in addition to reporting the roles of comorbidities [18]. Nevertheless, the effects of antiviral agents and AKI on the outcomes of patients with COVID-19 have not been reported in our previous article [18] and are still not fully understood. COVID-19 is an emergent disease with a limited number of studies in different aspects, especially on the comorbidities and complications. A previous systematic review and meta-analysis evaluated the prevalence of AKI in hospitalized COVID-19 patients in Iran [19]. However, previous studies did not evaluate the outcomes and effects of antiviral agents in hospitalized patients with COVID-19 who developed AKI. Therefore, this article aims to report the incidence of AKI in adult patients hospitalized with COVID-19 and to describe the associations between AKI, prescribing antivirals and mortality, admission to the intensive care unit (ICU), and prolonged hospitalization.

## Methods

### Study design and participants

It was a single-center retrospective study on adult patients with COVID-19 admitted to Baharloo Hospital in Tehran, Iran, from February 22 to April 19, 2020. The recruitment period for the study was similar to our previous published study [18].

Patients aged ≥18 years old, with a diagnosis of COVID-19 based on clinical presentations (i.e., fever, cough, dyspnea, myalgia and fatigue, hyposmia or anosmia, or hypogeusia or ageusia), radiographic features (i.e., bilateral or unilateral multifocal infiltration in chest plain radiography or ground-glass opacity in lung computed tomography scan), or a positive nasopharyngeal reverse transcription polymerase chain reaction (RT-PCR) for SARS-CoV-2 were included in this study. On the other hand, individuals with underlying immunodeficiency diseases affected by human immunodeficiency virus and/or acquired immunodeficiency syndrome or active cancer were excluded.

### Ethics statements

The study was performed in accordance with the Declaration of Helsinki. The Ethics Committee of Tehran University of Medical Sciences approved the study (Ethics Approval Code: IR.TUMS.VCR.REC.1399.228). The study was orally explained to the eligible patients, and we obtained written informed consent. All methods were carried out in accordance with the national guidelines and regulations. The authors did not have access to information that could identify individual participants during or after data collection.

### Data collection

The data were accessed for research purposes from February 22 to April 19, 2020. Data on patients’ demographics, medications, and underlying diseases were obtained from electronic medical records.

### Definitions

According to the Kidney Disease: Improving Global Outcomes (KDIGO) 2012 clinical practice guideline [20], AKI was defined as an increase in serum creatinine (SCr) ≥0.3 mg/dl (≥ 26.5 μmol/l) within 48 hours or ≥1.5 times increase in the baseline SCr which is occurred in last 7 days. We could not use the urine output criteria to define AKI as it was not regularly documented. Duration of hospitalization over the median (six days) was considered prolonged hospitalization. Antiviral treatment was defined for all patients who received one of the following treatments, including lopinavir/ritonavir (Kaletra), ribavirin, favipiravir, oseltamivir, remdesivir. Patients based on using antiviral medications or not and development of AKI or not were categorized into four groups: 1) without antiviral medications and without AKI development; 2) with antiviral medications and without AKI development; 3) without antiviral medications and with AKI development; and 4) with antiviral medications and with AKI development.

### Outcomes

In this study, mortality, admission to ICU, and prolonged hospitalization were considered outcomes.

### Statistical analysis

Continuous variables were expressed as mean (±standard deviation (SD)), and categorical ones were expressed as percentages. We performed an independent sample t-test for continuous variables and a chi-square test for categorical variables.

Also, univariable and multivariable logistic regression was utilized to determine the predictive effects of AKI and antiviral administration on composite outcomes, including mortality, ICU admission, and prolonged hospitalization. Variables with P<0.05 and missing data less than 30% in univariable analysis were included in multivariable analysis. Potential confounders including, age, sex, hypertension, diabetes, naproxen, indomethacin, corticosteroids, azithromycin, and treatment with one of other antibiotics (i.e., linezolid, vancomycin, carbapenem, piperacillin/tazobactam (tazocin), and cephalosporin). The results were represented as odds ratios (ORs), 95% CIs, and p-value.

Kaplan–Meier survival analysis by the log-rank was used to determine survival rates by AKI and antiviral medications. The level of significance for the p-value was considered 0.05. All analyses were performed using IBM SPSS version 24.0 software (IBM Corp., NY, USA).

## Results

### Baseline characteristics

**Fig 1:**
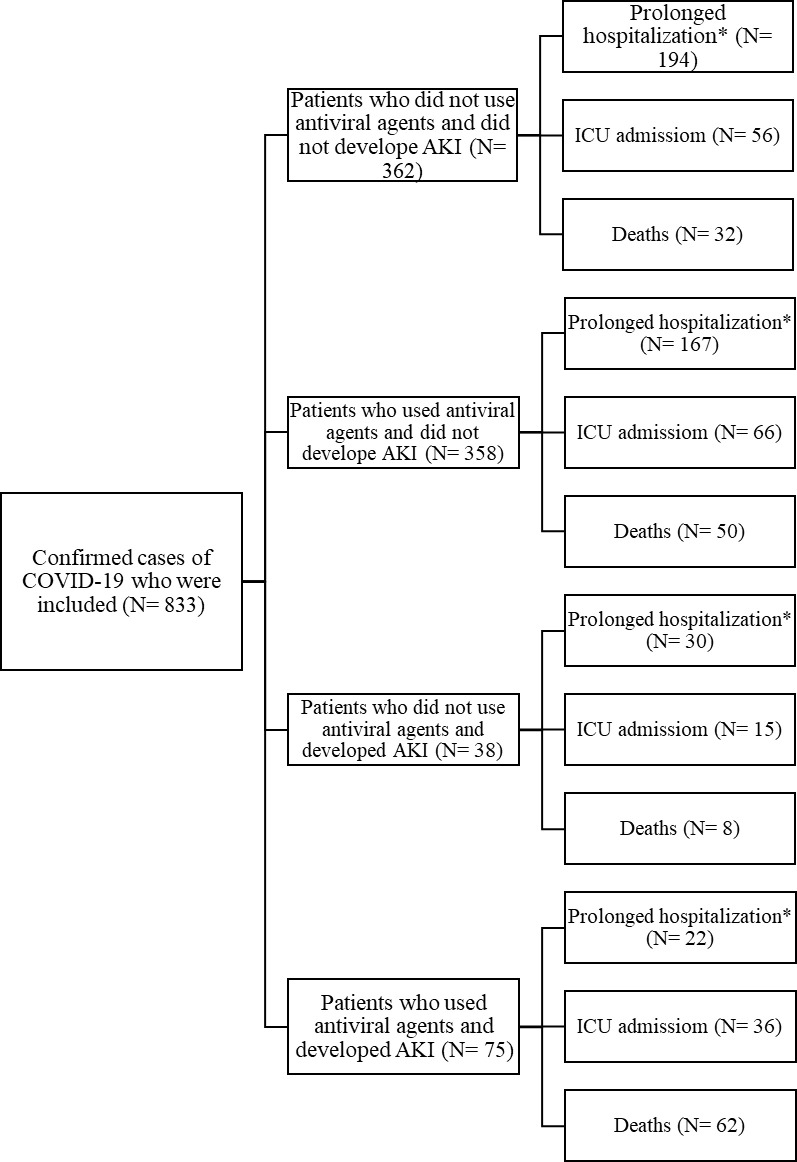
Flow diagram of study selection and outcomes. Abbreviations: COVID-19: coronavirus disease 2019; AKI: acute kidney injury; ICU: intensive care unit. *Duration of prolonged hospitalization defined as higher than median (six days).

Within the two-month period, 833 patients were included in this study (Fig 1). The participants were primarily male (54.4%) with a mean age of 55.29 (±17.58) years, mean BMI of 27.10 (±4.56) kg/m^2^, and baseline creatinine of 1.16 (±0.56) mg/dl. Hypertension (29.2%) followed by diabetes (26.2%) were the most common comorbidities among these patients, while chronic obstructive pulmonary disease (COPD)/asthma had the lowest frequency (5.4%). There was no significant difference in baseline characteristics and comorbidities between patients using antiviral agents and those without, except for gender (p = 0.002) (Table 1).

**Table 1:** Clinical characteristics of patients included in the study based on antiviral treatment.

Variables	All patients (N = 833)	Without antiviral medications (n = 400)	With antiviral medications (n = 433)	p-value
Age, mean years ±SD	55.29 ± 17.58	55.31± 18.39	55.27 ± 16.83	0.969
Mean BMI, mean kg/m2 ±SD	27.10 ± 4.65	26.68 ± 4.81	27.41 ± 4.51	0.091
First Creatinine, mean mg/dl ± SD	1.16 ± 0.56	1.14 ± 0.52	1.18 ± 0.59	0.342
Hospital stay, median day ±IQR	6 ± 6	6 ± 6	6 ± 6	0.318
**Gender**				
Women	380 (45.6)	205 (51.3)	175 (40.4)	0.002
Men	453 (54.4)	195 (48.8)	258 (59.6)	
**Comorbidities**				
Heart failure	127 (15.2)	66 (16.5)	61 (14.1)	0.333
Hypertension	243 (29.2)	116 (29)	127 (29.3)	0.917
Diabetes	218 (26.2)	99 (24.8)	119 (27.5)	0.370
COPD/Asthma	45 (5.4)	19 (4.8)	26 (6)	0.424
AKI incidence (%)	113 (13.6)	38 (9.5)	75 (17.3)	0.001

Abbreviations: BMI: body mass index; SD: standard deviation; IQR: interquartile range; COPD: chronic obstructive pulmonary disease; AKI: acute kidney injury.

*The definition of AKI is based on Kidney Disease: Improving Global Outcomes (KDIGO) 2012 clinical practice guideline.

After dividing the participants by administration of antiviral medications and incidence of AKI in four groups, there were significant differences between these four groups in age (p<0.0001), baseline creatinine (p<0.0001), gender frequency (p = 0.001), duration of hospital stay (p<0.0001), and preexisting hypertension (p = 0.002). The group that used antiviral medications and AKI occurred had the highest mean age (62.60 years), baseline creatinine (1.51 mg/dl), and duration of hospital stay (11 days). Also, the mentioned group had the highest proportion of underlying hypertension (45.3%) (Table 2).

**Table 2:** Clinical characteristics of patients included in the study based on antiviral treatment and incidence of acute kidney injury.

	Group 1 (n = 362)	Group 2 (n = 358)	Group 3 (n = 38)	Group 4 (n = 75)	p-value
Age, years ±SD	54.90 ± 18.09	53.73± 16.50	59.28± 20.89	62.60± 16.56	<0.0001
Mean BMI, kg/m2 ±SD	26.82± 4.91	27.53±4.57	25.51 ± 3.79	26.95± 4.31	0.165
First Creatinine, mg/dl ± SD	1.11 ± 0.51	1.11±0.58	1.45± 0.63	1.51±0.51	<0.0001
Hospital stay, day ±SD	6 ± 6	5 ± 5	7 ± 5	11 ±9	<0.0001
**Gender**					
Women	191 (52.8)	154 (43)	14 (36.8)	21 (28)	0.001
Men	171 (47.2)	204 (57)	24 (63.2)	54 (72)	
**Comorbidities**					
Heart failure	61 (16.9)	50 (14)	5 (13.2)	11 (14.7)	0.730
Hypertension	103 (28.5)	93 (26)	13 (34.2)	34 (45.3)	0.002
Diabetes	90 (24.9)	90 (25.1)	9 (23.7)	29 (38.7)	0.052
COPD/Asthma	18 (5)	20 (5.6)	1 (2.6)	6 (8)	0.689

Abbreviations: BMI: body mass index; SD: standard deviation; COPD: chronic obstructive pulmonary disease; AKI: acute kidney injury.

Group 1: Without AKI and Without antiviral medications (n = 362); Group 2: Without AKI and with antiviral medications (n = 358); Group 3: With AKI and Without antiviral medications (n = 38); Group 4: With AKI and with antiviral medications (n = 75).

Antiviral treatment was defined all patients who received one of following treatment: Lopinavir/Ritonavir (Kaletra), Ribavirin, Favipiravir, Oseltamivir, or Remdesivir.

*The definition of AKI is based on Kidney Disease: Improving Global Outcomes (KDIGO) 2012 clinical practice guideline.

### Medications

Out of 833 included patients, the most frequently utilized medication were hydroxychloroquine (84%), azithromycin (47.5%), and proton pump inhibitors (45.4%), whereas vancomycin (1.6%) and diphenhydramine (1.8%) had the lowest frequency. Between the four mentioned groups based on antiviral agents and AKI occurrence, there was a significant difference in the administration of linezolid (p<0.0001), carbapenem (p<0.0001), cephalosporin (p<0.0001), azithromycin (p<0.0001), naproxen (p<0.0001), indomethacin (p<0.0001), corticosteroids (p<0.0001), and piperacillin/tazobactam (p = 0.015). These medications, except for azithromycin, naproxen, and indomethacin, had the highest frequency in the group with antiviral medications and AKI incidence (Table 3).

**Table 3:** Prescribed medications in patients in different group of antiviral treatment and incidence of acute kidney injury.

Medications	Total (n = 833)	Group 1 (n = 362)	Group 2 (n = 358)	Group 3 (n = 38)	Group 4 (n = 75)	P-value
Linezolid	108 (13)	9 (2.5)	70 (19.6)	0	29 (38.7)	<0.0001
Vancomycin	13 (1.6)	3 (0.8)	5 (1.4)	2 (5.3)	3 (4)	0.051
Carbapenem	67 (8)	14 (3.9)	35 (9.8)	3 (7.9)	15 (20)	<0.0001
Piperacillin/Tazobactam (Tazocin)	37 (4.4)	9 (2.5)	18 (5)	2 (5.3)	8 (10.7)	0.015
Cephalosporin	99 (11.9)	49 (13.5)	24 (6.7)	8 (21.1)	18 (24)	<0.0001
Hydroxy Chloroquine	700 (84)	309 (85.4)	296 (82.7)	33 (86.8)	62 (82.7)	0.991
Azithromycin	396 (47.5)	207 (57.2)	137 (38.3)	28 (73.7)	24 (32)	<0.0001
Naproxen	320 (38.4)	184 (50.8)	86 (24)	23 (60.5)	27 (36)	<0.0001
Indomethacin	82 (9.8)	17 (4.7)	58 (16.2)	2 (5.3)	5 (6.7)	<0.0001
Diphenhydramine	15 (1.8)	7 (1.9)	5 (1.4)	1 (2.6)	2 (2.7)	0.842
PPIs	378 (45.4)	155 (42.8)	169 (47.2)	15 (39.5)	39 (52)	0.347
Statins	129 (15.5)	61 (16.9)	44 (12.3)	8 (21.1)	16 (21.3)	0.102
ACEIs_ARBs	82 (9.8)	38 (10.5)	29 (8.1)	4 (10.5)	11 (14.7)	0.336
Corticosteroid	89 (10.7)	35 (9.7)	33 (9.2)	1 (2.6)	20 (26.7)	<0.0001

Abbreviations: AKI: acute kidney injury; PPIs: Proton-pump inhibitors; ACEIs: Angiotensin-converting enzyme inhibitors; ARBs: Angiotensin II receptor blockers.

Group 1: Without AKI and Without antiviral medications (n = 362); Group 2: Without AKI and with antiviral medications (n = 358); Group 3: With AKI and Without antiviral medications (n = 38); Group 4: With AKI and with antiviral medications (n = 75).

Antiviral treatment was defined all patients who received one of following treatment: Lopinavir/Ritonavir (Kaletra), Ribavirin, Favipiravir, Oseltamivir, or Remdesivir.

### Outcomes

Overall, the percent of prolonged hospitalization, ICU admission, and mortality were 54.4%, 20.8%, and 13.4%, respectively. There were significant differences in all the mentioned outcomes between the four groups in which the group including patients who developed AKI and used antiviral agents had the most significant proportion of the composite outcome (p<0.0001) (Table 4). Also, patients with AKI and without administration of antivirals had a higher rate of the composite outcome (Table 4 and Fig 1).

**Table 4:** Mortality, intensive care unit admission and prolonged hospitalization of patients included in the study based on different categories of antiviral treatment and incidence of acute kidney injury.

	All patients (N = 854)	Group 1 (n = 362)	Group 2 (n = 358)	Group 3 (n = 38)	Group 4 (n = 75)	P-value
Mortality	112 (13.4)	32 (8.8)	50 (14)	8 (21.1)	22 (29.3)	<0.0001
ICU admission	173 (20.8)	56 (15.5)	66 (18.4)	15 (39.5)	36 (48)	<0.0001
prolonged hospitalization (higher than median = 6 days)	453 (54.4)	194 (53.6)	167 (46.6)	30 (78.9)	62 (82.7)	<0.0001

Abbreviations: ICU: intensive care unit.

Group 1: Without AKI and Without antiviral medications (n = 362); Group 2: Without AKI and with antiviral medications (n = 358); Group 3: With AKI and without antiviral medications (n = 38); Group 4: With AKI and with antiviral medications (n = 75).

**Fig 2:**
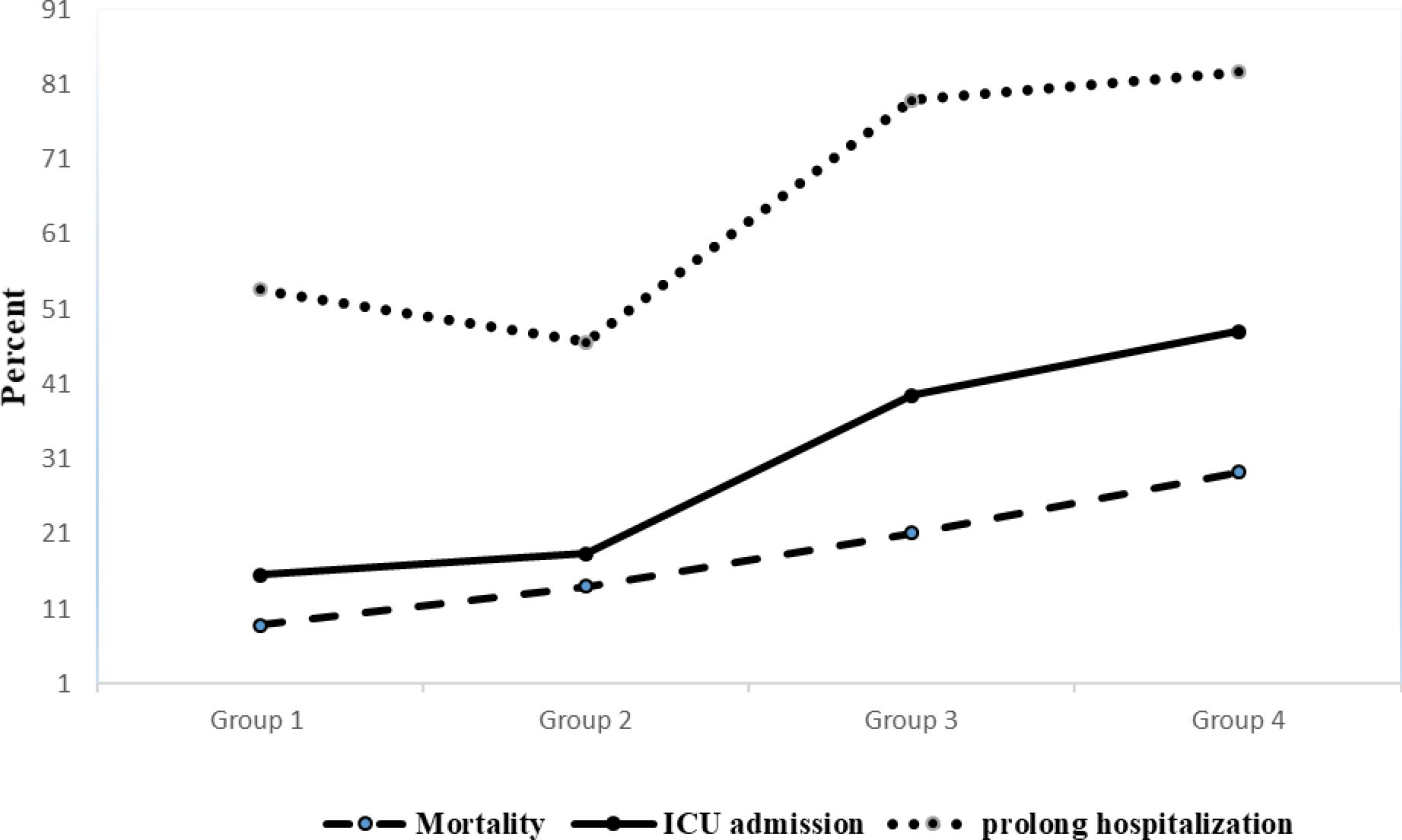
Percent of main outcome in patients by different category of nephrotoxic treatment and incidence of acute kidney injury (AKI). (Group 1: Without AKI and without antiviral medications (n = 362); Group 2: Without AKI and with antiviral medications (n = 358); Group 3: With AKI and without antiviral medications (n = 38); Group 4: With AKI and with antiviral medications (n = 75)).

**Fig 3:**
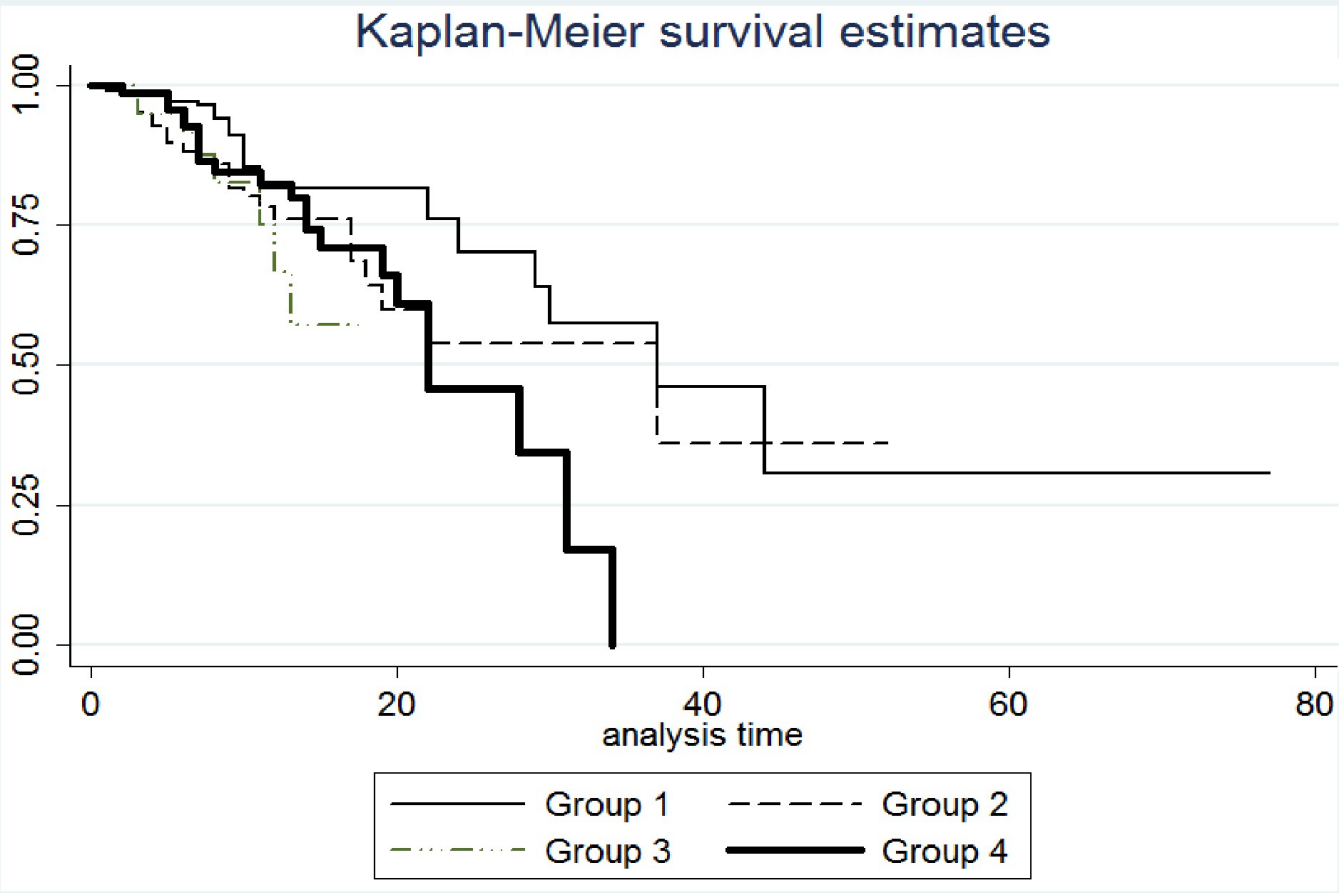
Kaplan Maier survival time of patients based on different category of antiviral treatment and incidence of acute kidney injury (AKI). (Group 1: Without AKI and Without antiviral medications (n = 362); Group 2: Without AKI and with antiviral medications (n = 358); Group 3: With AKI and without antiviral medications (n = 38); Group 4: With AKI and With antiviral medications (n = 75)).

Our multivariable logistic regression showed that AKI had a more prominent effect on mortality and ICU admission as patients with AKI and without using antiviral medications compared to those without AKI and with using antivirals had more OR of ICU admission (5.87; 95% CI: 3.75, 9.18 vs. 2.69; 95% CI: 1.33, 5.44) and mortality (5.60; 95% CI: 3.31, 9.47 vs. 2.10; 95% CI: 0.88, 4.99). On the other hand, antiviral agents had a greater effect than AKI on length of hospitalization (OR = 3.38; 95% CI: 1.75, 6.52 in patients with antivirals and without AKI; OR = 1.24; 95% CI: 0.86, 1.80 in patients with AKI and without antivirals) (Table 5). Figs 2 and 3 represent the Kaplan-Maier curves of the survival rate and survival time of patients in different categories based on antiviral agents and AKI occurrence, respectively.

**Table 5:** Logistic regression and odds ratio for different category of antiviral treatment and incidence of acute kidney injury and mortality, intensive care unit admission and prolonged hospitalization.

**Outcome: Death**				
	Age and sex adjusted odds ratio (95% CI)	P-value	Multivariate adjusted odds ratio (95% CI) *	P-value
Without antiviral and without AKI	1	1	1	
With antiviral and without AKI	2.08 (0.88–4.91)	0.081	2.10 (0.88–4.99)	0.074
Without antiviral and with AKI	6.19 (3.73–10.28)	<0.0001	5.60 (3.31–9.47)	<0.0001
With antiviral and with AKI	8.68 (4.35–15.30)	<0.0001	7.89 (3.85–16.17)	<0.0001
**Outcome: ICU Admission**				
Without antiviral and without AKI	1	1	1	
With antiviral and without AKI	2.96 (1.49–5.88)	0.002	2.69 (1.33–5.44)	0.005
Without antiviral and with AKI	7.18 (4.67–11.04)	<0.0001	5.87 (3.75–9.18)	<0.0001
With antiviral and with AKI	19.22 (9.98–37.02)	<0.0001	15.22 (8.06–27.32)	<0.0001
**Outcome: prolonged hospitalization (higher than median)**				
Without antiviral and without AKI	1	1	1	
With antiviral and without AKI	3.59 (1.88–6.84)	<0.0001	3.38 (1.75–6.52)	<0.0001
Without antiviral and with AKI	1.51 (1.06–2.12)	<0.0001	1.24 (0.86–1.80)	0.240
With antiviral and with AKI	5.91 (2.72–11.84)	<0.0001	4.82 (2.18–8.66)	<0.0001

Abbreviations: ICU: intensive care unit; AKI: acute kidney injury; CI: confidence interval.

*model adjusted for confounders age, sex, hypertension, diabetes, naproxen, indomethacin, corticosteroids, azithromycin and treatment with at least other antibiotics (linezolid, vancomycin, carbaoenem, piperacillin/tazobactam (tazocin), cephalosporin).

## Discussion

According to our findings, the incidence of AKI was significantly higher in those who utilized antivirals. Moreover, those with AKI and administration of antivirals were older and had a significantly higher rate of underlying hypertension. Our findings also represented that COVID-19 patients on antiviral medications who developed AKI had increased odds of mortality and ICU admission more than those without using antiviral medications and without the development of AKI. Nevertheless, the effects of AKI on mortality and ICU admission were more prominent than antivirals. Considering the length of hospitalization as our outcome, we revealed that antiviral medications are more associated with prolonged hospitalization than AKI.

Our findings showed that the incidence of AKI was almost two times more common in patients using antivirals than those who did not (17.3% vs. 9.5%; p = 0.001). In this regard, a multicenter observational study on 415 COVID-19 patients in France and Belgium showed that there is a significant difference in utilization of lopinavir/ritonavir and development of AKI (p = 0.03), and those patients had the highest rate of need for renal replacement therapy (RRT) [13]. There are different mechanisms, including transporter defects, crystal deposition, vascular injury, apoptosis, and mitochondrial injury, that have been proposed for antiviral-induced nephrotoxicity [21]. Of note, COVID-19, through direct viral invasion, endothelial dysfunction, renin-angiotensin system dysregulation, cytokine storm, microvascular thrombosis, and other systemic effects, can develop AKI [22, 23].

In the article by Chan et al., preexisting hypertension with a frequency of 38% had the highest frequency among comorbidities, and it was significantly higher in COVID-19 patients with AKI than those without AKI (45% vs. 33%; p<0.001) [24]. In this regard, only hypertension was significantly different among our included participants which were divided into four groups by AKI and antiviral medication status (p = 0.002).

A single-center cohort study on 158 deceased Mexican patients due to COVID-19 revealed that azithromycin (60.6%) followed by hydroxychloroquine (53.0%) had the highest frequency of administration in these patients [25], while hydroxychloroquine was prescribed more commonly among all patients in our center. This discrepancy might be due to the difference in using off-label drugs in various countries [25].

We found that AKI occurrence, antiviral agents, and both of them were associated with our composite outcome and the effects of AKI was greater than antivirals. In accordance with our results, the study by Teoh et al. on 814 patients affected by SARS-CoV-2 represented that administration of antiviral medications (OR = 11.06 (95% CI: 2.66, 45.98), corticosteroids (OR = 3.31; 95% CI: 1.66, 6.62), baseline creatinine (OR = 1.02; 95% CI: 1.01, 1.03), and preexisting hypertension (OR = 3.03; 95% CI: 1.63, 5.62) were associated with AKI [26]. Consequently, the AKI was associated with their composite endpoint (i.e. ICU admission, invasive mechanical ventilation, and death) (OR = 2.44; 95% CI: 1.06, 5.58) [26]. A systematic review and meta-analysis of 15 studies including 3615 patients showed that AKI was associated with increased mortality (relative risk (RR) = 13.38; 95% CI: 8.15, 21.95) and ICU admission (RR = 5.90; 95% CI: 1.32, 26.35) [27]. Furthermore, a meta-analysis of 5528 patients with COVID-19 showed a significantly strong association between AKI and death (OR = 12.1; 95% CI: 2.3, 62.4) and adverse outcomes (OR = 5.1; 95% CI: 1.8, 14.8) [28]. The article by Grimaldi et al. showed no significant difference in ICU survival rate among patients without antiviral medications and those who used lopinavir/ritonavir, hydroxychloroquine, and other antiviral agents (p = 0.22) and the ICU survival rate was significantly greater in those without AKI (p<0.001) [13]. It should be noted that this study only included COVID-19 patients with moderate-to-severe acute respiratory distress syndrome [13]. Moreover, a systematic review and meta-analysis of five studies, which divided patients by severity, showed that severe patients with COVID-19 had higher odds of AKI occurrence and administration of antiviral agents than the non-severe group [29]. A meta-analysis of five randomized controlled trials (RCTs) including 3095 participants showed no significant increased risk of AKI in COVID-19 patients on remdesivir therapy (RR = 0.71; 95% CI: 0.43, 1.18) [30]. An analysis on the US Food and Drug Administration database including 12869 patients with COVID-19 showed remdesivir increased the risk of AKI by 2.81 times (95% CI: 2.48, 3.18) [31]. However, over time and during the third quarter of 2022, the reporting OR of AKI in patients with COVID-19 using remdesivir decreased (reporting OR: 1.50; 95% CI 0.91, 2.45) [32]. These findings are in a similar way to our results which showed a higher impact of AKI than antiviral medications on composite outcomes.

Our retrospective study had some limitations, including: 1) the effects of each antiviral medication were not evaluated on outcomes; 2) non-measured confounding factors might exist and interfere with the findings, although we tried to adjust for many known confounders; 3) we did not collect any severity scores such as pneumonia severity index (PSI) [33], Charlson comorbidity index [34], and A-DROP [35]; 4) We not only included patients with positive RT-PCR for SARS-CoV-2 but also included patients based on imaging and clinical manifestations which are not the gold standard of diagnosis, so some non-COVID-19 patients might be included [36]; 5) This was a single center study which most of the participants were from Iran, so these results cannot be generalized to other nations, especially due to racial and/or ethnic disparities of COVID-19 occurrence and severity [37, 38]; 6) Selection bias is an inevitable part of this study because it is not a RCT; 7) There is the probability of sampling bias because of using convenience sampling method.

## Conclusions

The occurrence of AKI in COVID-19 patients who used antiviral agents is associated with severity and death. AKI development is more important in mortality and ICU admission despite antivirals that have much effect on the duration of hospitalization. Prevention from AKI development in the patients using antivirals should be considered. Further large-scale RCTs might be needed to clarify the associations.

## List of abbreviations

ACEIs: Angiotensin-converting enzyme inhibitors
AKI: acute kidney injury
ARBs: Angiotensin II receptor blockers
ARDS: acute respiratory distress syndrome
BMI: body mass index
CI: confidence interval
COPD: chronic obstructive pulmonary disease
COVID-19: coronavirus disease 2019
ICU: intensive care unit
IQR: interquartile range
KDIGO: Kidney Disease: Improving Global Outcomes
OR: odds ratio
PPIs: Proton-pump inhibitors
PSI: pneumonia severity index
RCT: randomized controlled trial
RR: relative risk
RRT: renal replacement therapy
RT-PCR: reverse transcription polymerase chain reaction
SARS-CoV-2: severe acute respiratory syndrome coronavirus 2
SCr: serum creatinine
SD: standard deviation
UK: United Kingdom
US: United States
WHO: World Health Organization
